# AI-based predictive approach via FFB propagation in a driven-cavity of Ostwald de-Waele fluid using CFD-ANN and Levenberg–Marquardt

**DOI:** 10.1038/s41598-024-60401-2

**Published:** 2024-05-14

**Authors:** Ahmed Refaie Ali, Rashid Mahmood, Atif Asghar, Afraz Hussain Majeed, Mohamed H. Behiry

**Affiliations:** 1 Department of Mathematics and Computer Science, Faculty of Science, Menoufia University, Shebin El Kom, 32511 Menofia Egypt; 2 Department of Mathematics, Air University, PAF Complex E-9, Islamabad, 44000 Pakistan; 3 School of Energy and Power Engineering, Jiangsu University, Zhenjiang, 212013 China; 4 Department of Artificial Intelligence, Faculty of Artificial Intelligence, Egyptian Russian University, Badr City, 11829 Egypt

**Keywords:** Artificial intelligence (AI), Artificial neural networks (ANNs), Driven cavity, Finite element method (FEM), Ostwald de-Waele fluid, Hybrid CFD-ANN, Levenberg Marquardt Algorithm, Applied mathematics, Computational science, Computer science, Information technology, Fluid dynamics, Information theory and computation

## Abstract

The integration of Artificial Intelligence (AI) and Machine Learning (ML) techniques into computational science has ushered in a new era of innovation and efficiency in various fields, with particular significance in computational fluid dynamics (CFD). Several methods based on AI and Machine Learning (ML) have been standardized in many fields of computational science, including computational fluid dynamics (CFD). This study aims to couple CFD with artificial neural networks (ANNs) to predict the fluid forces that arise when a flowing fluid interacts with obstacles installed in the flow domain. The momentum equation elucidating the flow has been simulated by adopting the finite element method (FEM) for a range of rheological and kinematic conditions. Hydrodynamic forces, including pressure drop between the back and front of the obstacle, surface drag, and lift variations, are measured on the outer surface of the cylinder via CFD simulations. This data has subsequently been fed into a Feed-Forward Back (FFB) propagation neural network for the prediction of such forces with completely unknown data. For all cases, higher predictivity is achieved for the drag coefficient (CD) and lift coefficient (CL) since the mean square error (MSE) is within ± 2% and the coefficient of determination (R) is approximately 99% for all the cases. The influence of pertinent parameters like the power law index (n) and Reynolds number (*Re*) on velocity, pressure, and drag and lift coefficients is also presented for limited cases. Moreover, a significant reduction in computing time has been noticed while applying hybrid CFD-ANN approach as compared with CFD simulations only.

## Introduction

Artificial Neural Networks (ANNs) have transformed machine learning and have been leading among many recent breakthroughs in artificial intelligence. Their capacity to learn from data and produce to unseen, new examples that makes it a powerful tool for solving complex problems and functions. Artificial neural networks (ANNs) are used in various domains that include processing of natural languages, computer vision, automatic vehicles, speech identification, exhortation systems, health care, accounting, and finance and many more.

As artificial neural network (ANNs) is supposed to be a class of models influenced by the system and mechanism of the human brain. ANN made up of mutually dependent nodes (neurons) arranged in layers. For modelling performance limitation of artificial neural network can be powerful tool. The outcome of the artificial neural networks can help experts of energy to construct the model with a high-level execution, responsibility, and strength and with a minimum inconstancy^1–4^.

In many applications, ANN models have been employed to predict process outputs to reduce computational costs associated with the simulations. An important class of neural networks that are trained to solve supervised learning problems based on physical laws governed by partial differential equations namely Physics-Informed Neural Networks (PINNs), refer to^5,6^ for more details. Physics is imposed through differential equations in PINNs, for instance through the Navier–Stokes equations in flow problems. The ANN model by Jassim et al.^7^ has proven highly effective in forecasting the thermal–hydraulic characteristics of a flat tube bank arranged in line. Employing the ANN methodology for predicting thermal-fluid attributes shows notable concurrence with simulation findings. As a result, they advocate for this technique as a valuable resource, given its capacity to deliver prompt, dependable, and precise outcomes. Additionally, it furnishes preliminary approximations that can significantly aid engineers in tackling intricate challenges related to fluid dynamics.

Mahmood et al.^8^ have proposed a model for $2D$ unsteady flow of power law fluid in a domain. For the training and validation of the model CFD results (FEM based) have been used to predict hydrodynamics forces with no more simulations to avoid computational cost. Gunipar et al.^9^ also employed machine learning regression and neural-network methods to develop a mathematical model. This model was trained using the drag coefficient dataset obtained from CFD simulations.

Researchers have tried to examine nonlinear fluid flowing across obstacles and computing the hydrodynamic forces including the drag and lift. For the analysis of hydrodynamics forces different fluid flow configurations of engineering interest have been used. Stream properties and their control are also being studied over many bluff bodies. It is also worth noting that the location of impediments in crossflow serves a real purpose and performs a crucial role. Research^10–15^ reviewed a great deal on non-Newtonian flow of fluid around one cylinder. Bharti et al.^16^ analyzed the steady flow behaviour of power law fluids as they pass around an unconfined circular cylinder. That research delved into how the Reynolds number and power law index impact flow characteristics, including streamline profiles, vorticity, and surface pressure.

There is small amount of study in research on incompressible power-law liquid running across circular cylinders in tandem configuration. Many investigations in fluids that are non-Newtonian have been conducted on the location of the double containers^17–20^. Zhang et al.^21^ worked unexpected consequences like impeller fractures and structural damage to the entire pumping system might result from high alternating stress, unstable pressure waves and extreme vibration. Alam et al.^22^ studied the features of flowing pattern and the influence of liquid forces on two spaced cylinders. There has been a lot of computer work done to explore hydrodynamics forces for barriers in the Newtonian flow, however studying the impact of complex viscous law on drag and lift remains in its infancy. The researchers assert that a range of modelling techniques may be used with a mathematical fluid dynamics solver to determine the effects of movement shapes, including the velocity of main swirl movement and the time of second container contact, for circulation between two rows of separated containers. The Reynolds number and intervals separation pattern for the flow across next to cylinders may be determined via numerical analysis^23–26^.

Majeed et al.^27^ examined numerically the laminar flow in a cavity using FEM. Simulations of this fluid flow involves solving the Navier–stokes equations and the Power law model within a finite element domain. Ain et al.^28^ investigated the control effects by using a passive device. They have used the passive device in two different ways. Finite element method calculations can provide valuable appreciation into the effectiveness of passive control methodologies for dealing hydrodynamic forces around circular cylinders in unsteady flow situation.

Mahmood et al.^29^ investigated the properties of flow of modified Cross model (MCM) using finite element method. The hydrodynamic forces are computed for a variety of parameters involved. In another paper, Mahmood et al.^30^ considered $2D$ unsteady flow of an incompressible fluid over a circular obstacle in a domain using power law and concluded, there are different trajectories using various values of $n=0.5, 1 and 1.5.$

Over the past decade, AI-based algorithms have made remarkable progress, leading to the standardization of several methods in CFD simulations (Smith et al.^31^; Brown and Williams^32^; Anderson and Wilson^33^). One of the most promising developments in this domain is the coupling of CFD with artificial neural networks (ANNs) to predict fluid forces generated when a fluid interacts with obstacles within a flow domain (Johnson and Davis^34^; Roberts and White^35^). This hybrid approach, often incorporating the Levenberg–Marquardt algorithm, offers a significant reduction in computational resource requirements, including memory and time considerations (Parker and Moore^36^; Adams and Johnson^37^). Such advancements hold the potential to revolutionize the field of fluid dynamics, enabling the efficient prediction of essential parameters like drag coefficient ${C}_{D}$ and lift coefficient ${C}_{L}$ even in scenarios involving non-linear rheological relations and complex flow behaviour (Smith and Taylor^38^; Wilson and Brown^39^). This introduction sets the stage for exploring the implications and findings of data-driven techniques, particularly ANNs, in the context of fluid dynamics, laying the foundation for future applications in three-dimensional and turbulent flows (Davis and Anderson^40^). The recent works^41–48^ cover a wide range of topics in physics and engineering, including plasma physics, fluid dynamics, nonlinear equations, and heat transfer. They explore various aspects of these fields and provide insights into the behaviour of different physical systems.

In the current investigation, the hybrid CFD-ANN approach has been followed that represents essential features of fluid dynamics, numerical modelling, and engineering analysis. By combining CFD and ANN, we were able to demonstrate a significant decrease in both memory and time requirements for computational work involving temporal derivatives. These interesting values have been estimated using CFD simulations, and those computations have been used as the foundation for both the training and evaluation of artificial neural networks using these data sets.

## Physical configuration and governing laws

**Figure 1:**
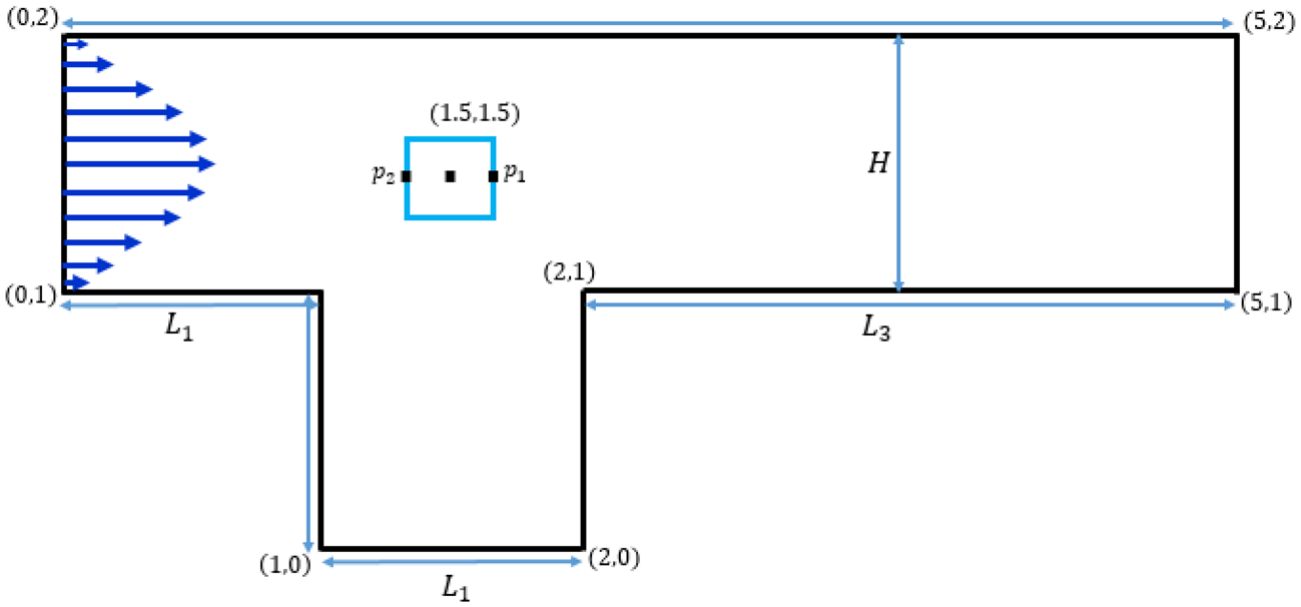
Schematic representation of the flow domain.

Consider the schematic representation of a square cavity attached with a channel illustrated in Fig. 1. This is an important benchmark configuration that combines the flow features in a channel and of cavity dynamics. The dimensions of the domain have been represented by showing the coordinates at the vertices. An obstacle with side D = 0.1 m has been installed in the domain at the position (1.5 m, 1.5 m). At the inlet a fully developed parabolic velocity is given with a maximum value of ${U}_{max}$ that is the controlling parameter for Reynolds number. A choice of *Re* with magnitude of 20 and 100 was made to switch between the stationary and non-stationary regimes.

The following is an expression of the conservation laws that apply to 2D incompressible, isothermal and time dependent flows.

1 $$\frac{{\partial u_{i} }}{{\partial x_{i} }} = 0, \,$$

2 $$\frac{\partial {u}_{i}}{\partial t}+\frac{\partial }{\partial {x}_{j}}\left({u}_{i}{u}_{j}\right)+\frac{1}{\rho }\frac{\partial p}{\partial {x}_{i}}=\frac{1}{\rho }\frac{\partial }{\partial {x}_{j}}\left[\mu \left(\dot{\gamma }\right)\left(\frac{\partial {u}_{i}}{\partial {x}_{j}}+\frac{\partial {u}_{j}}{\partial {x}_{i}}\right)\right], i,j=1\dots 2$$

The relationship that represents a change in viscosity based on Ostwald de-Waele model aka Power Law (PL) fluid with the shear rate is written as follows:

3 $$\mu (\dot{\gamma }) = m(\dot{\gamma })^{n - 1} {, }$$

where *m* represents consistency coefficient; *n* is the power law index; $\dot{\gamma }$ is the magnitude of shear rate. The boundary conditions at various parts of the domain are given as

At inlet: $$u = 4U_{\max .} y(H - y)/H^{2}$$, $$v = 0$$,

At outlet: $$p=0$$,

At Walls and on obstacle: $u = v = 0$.

The formula for determining the involved $Re$ for the power law fluid model is as follows:

4 $$Re=\frac{\rho {\left({U}_{mean}\right)}^{2-n}{D}^{n}}{\mu }$$

meanings of all the parameters correspond to their standard assumptions. It should be noticed that the temporal derivative in Eq. (2) is set to zero for lower values of Re. The calculation of the drag and lift forces acting on the cylinder involves the following line integrals.

5 $$\left\{ {\begin{array}{*{20}c} {F_{D} = \int\limits_{c} {\left( {\sigma .n} \right)e_{x} ds} } \\ {F_{L} = \int\limits_{c} {\left( {\sigma .n} \right)e_{y} ds} } \\ \end{array} } \right.$$

where $\sigma$ is the Cauchy stress tensor, and *n* is the unit normal vector. Normalizing the drag and lift forces yields us to their corresponding dimensionless coefficients as

6 $$C_{D} = \frac{{2F_{D} }}{{\rho U_{mean}^{2} D}}, \, C_{L} = \frac{{2F_{L} }}{{\rho U_{mean}^{2} D}}{,}$$

where $U_{mean}$ represents the average velocity of the parabolic inflow profile.

## Hybrid CFD-ANN scheme

### CFD simulations-generation of training data sets

The model partial differential equations along with the rheological law representing Ostwald de-Wale PL fluid (1–3) have been simulated using commercial finite element-based solver COMSOL by a suitable choice of elements from the available library to approximate the velocity and pressure values approximations. Newton's approach is utilized to solve discrete non-linear algebraic systems, and a direct solver PARDISO is adopted as inner linear solver. The following convergence condition is set for the nonlinear iteration.

$$\left| {\frac{{\Lambda^{n + 1} - \Lambda^{n} }}{{\Lambda^{n + 1} }}} \right| < 10^{ - 6} { ,}$$

where $\Lambda$ indicates a component of the solution vector.

**Figure 2:**
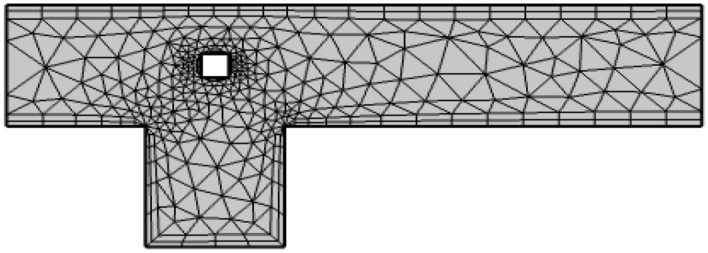
The computational grid at coarse level.

Figure 2 depicts the coarse computational grid used for the present study. For the accurate computation of quantities of interest including the drag and lift coefficients, the grid is more refined near the obstacle. Although meshing is performed at many different levels to optimally divide the domain into enough finite elements, only the coarsest level is shown here.

Table 1 contains an enumeration of mesh statistics at various Refinement Levels (RL). The number of elements (EL) and related global degrees of freedom (DOF) for velocity and pressure data are shown. The table demonstrates that the minimum number of elements (769) and degrees of freedom (4032) are available at level 1, while the largest number of elements (52,844) and degrees of freedom (250,024) are available at level 9 for the collection of data.

**Table 1:** Number of degrees of freedom for different refinement levels.

RL	# EL	# DOF
${RL}_{1}$	769	4032
${RL}_{2}$	1167	6130
${RL}_{3}$	1786	9245
${RL}_{4}$	2962	15,171
${RL}_{5}$	3590	21,648
${RL}_{6}$	6707	33,213
${RL}_{7}$	16,139	78,925
${RL}_{8}$	39,724	191,180
${RL}_{9}$	52,844	250,024

Table 2 shows that code validation under same geometric and parametric settings as in^48^, which validates the existing code.

**Table 2:** Code validation for ${C}_{D}$ and ${C}_{L}$ at Re = 20.

Drag and lift coefficients	Schaefer and Turek^48^	Present work
${C}_{D}$	5.5785	5.5785
${C}_{L}$	0.0106	0.0106

In Table 3, the variation in numerical data for ${C}_{D}$ and ${C}_{L}$ at all levels of refinements is shown to show the grid convergence and sufficiency of the underlying grid. Since the results at refinement levels 8 and 9 only differ by less than 1% so to save computational cost, all further simulations have been performed on level 8 of refinement. It is worth mentioning that a negative ${C}_{L}$ indicates upward lift forces are playing a more significant role that the downward forces.

**Table 3:** Grid Convergence for Re = 20 and n = 1.

Refinement level	${C}_{D}$	${C}_{L}$
${RL}_{1}$	4.9067	− 3.403E−1
${RL}_{2}$	4.8622	− 3.3395E−1
${RL}_{3}$	4.8091	− 3.366E−1
${RL}_{4}$	4.7846	− 3.283E−1
${RL}_{5}$	4.7731	− 3.251E−1
${RL}_{6}$	4.7689	− 3.246E−1
${RL}_{7}$	4.7642	− 3.241E−1
${RL}_{8}$	4.7624	− 3.230E−1
${RL}_{9}$	4.7624	− 3.228E−1

### Construction of ANN

**Figure 3:**
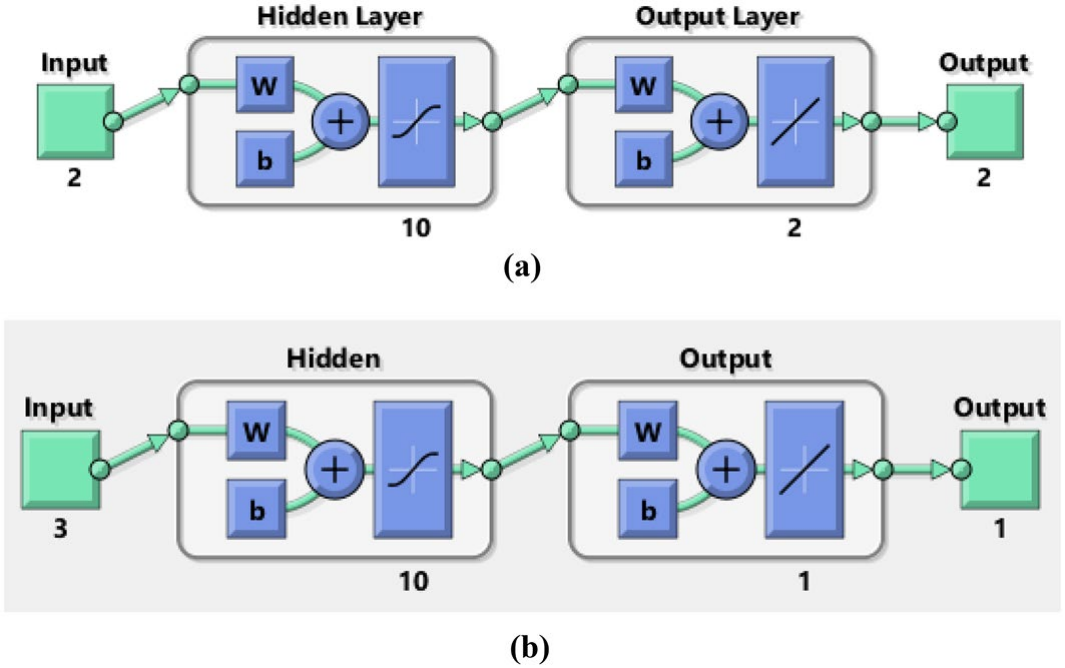
(**a**) Neural network block diagram for stationary case. (**b**) Neural network block diagram for time dependent case.

To implement machine learning algorithms, an artificial neural network has been created using multilayers namely input, hidden and output layers. The schematic diagram of underlying ANN is presented in Fig. 3a,b. The underlying ANN model consists of 2 input layers (for n and Re), 2 output layers (for drag and lift coefficients) and 10 hidden layers for the stationary case while for the time dependent case the number of input layers is increased to 3 to include time-step size in the input parameters. For all cases, 70% data is used for training phases while 15% each for testing and validation phases respectively. To minimize the loss function Levenberg–Marquardt optimization algorithm has been employed with suitable activation functions such as TANSIG (f(x)) and PURELIN (g(x)) in the hidden and output layers respectively. These activation functions can be represented mathematically as $f\left( x \right) = \frac{2}{{1 + e^{ - 2x} }} - 1$ and ${\text{g}}\left( x \right) = x$. LM algorithms works based on feed-forward and back-propagation and computes the gradient of Loss function w.r.t the weights in the neural network. These gradients are then used to update the weights using some optimization algorithm in the training step.

## Results and discussion

### Stationary case *Re* = *20*

By performing line integration over the boundary of the obstacle as provided in Eq. (5), drag and lift forces have been computed and subsequently their non-dimensional analogue, the drag and lift coefficients ${C}_{D}$ and ${C}_{L}$ respectively. Table 4 display the fluctuations that occur in standard hydrodynamic quantities, such as the coefficients of ${C}_{D}$ and ${C}_{L}$ defined in Eq. (6). It is discovered that the ${C}_{D}$ and ${C}_{L}$ increase with the power law index. Furthermore, lift forces are shown with negative values of ${C}_{L}$, which translates to a predominance of upward-directed lift force. When impediments are positioned directly above the cavity region, fluid is forced downward into the cavity, creating upward thrust on the obstruction, leading to negative values for the lift coefficient. The maximum ${C}_{D}$ and ${C}_{L}$ of 6.4225 and − 0.5996 are achieved, respectively, based on the computed values over the obstacle having its centre placed at (1.5, 1.5). For small values of *n*, the drag force decreases because of the fluid's viscosity decreasing with shear rate and the power law behaving as a shear thinning material, but for large values of *n*, the fluid's viscosity increasing with the rate of deformation leading to an increase in the drag coefficient.

**Table 4:** ${C}_{D}$ and ${C}_{L}$ coefficient with *Re* = *20* for different $n.$

${\text{n}}$	${C}_{D}$	${C}_{L}$
$0.3$	$3.032584977$	− 5.9575782E−2
$0.5$	$3.484985563$	− 1.48658415E−1
$0.7$	$3.991902453$	− $2.20603492$E−1
$0.9$	$4.508078988$	− $2.88375439$E−1
$1.0$	$4.762300406$	− $3.22801126$E−1
$1.1$	$5.012757972$	− $3.58097104$E−1
$1.3$	$5.500213963$	− $4.32297397$E−1
$1.5$	$5.965779351$	− $5.12749321$E−1
$1.7$	$6.422493958$	− $5.99570335$E−1

Change in pressure measured at the front and back of the obstacle as a function of *n* at Re = *20* is shown in Table 5. Based on the obtained numerical data, it is determined that the pressure drop increases as *n* grows larger. Because a power law fluid exhibits the characteristics of a shear-thinning fluid when n is less than one, a Newtonian fluid when n is equal to one, and elucidates the properties of a shear-thickening fluid, the viscosity of a power law fluid increases when the magnitude of n is increased. As a result, the fluid strikes the obstacle with more force, which causes the pressure drop to increase.

**Table 5:** Pressure drop with Re = 20 for various $n$.

$n$	$\delta p={p}_{2}-{p}_{1}$
0.3	0.072315351
0.5	0.075156883
0.7	0.078410314
0.9	0.081509774
1.0	0.082965494
1.1	0.084353817
1.3	0.086935280
1.5	0.089298733
1.7	0.091514916

Table 6 explains the range of values for the ${C}_{D}$ and ${C}_{L}$ when encountering a square obstacle cantered at coordinates (1.5, 1.5). As the Re increases, the ${C}_{D}$ and ${C}_{L}$ drop. Furthermore, lift forces are shown with negative values of ${C}_{L}$, which translates to a predominance of upward-directed lift force. When impediments are positioned directly above the cavity region, fluid is forced downward into the cavity, creating upward thrust on the obstruction, leading to negative values for the ${C}_{L}$.

**Table 6:** ${C}_{D}$ and ${C}_{L}$ with $n=1$ for various $Re.$

Re	${C}_{D}$	${C}_{L}$
1	40.700034	− 3.421546E0
5	10.490174	− 1.382850E0
10	6.804359	− 6.483126E−1
15	5.476226	− 4.294371E−1
20	4.762300	− 3.228011E−1
25	4.306980	− 2.567965E−1
30	3.987982	− 2.098634E−1
35	3.751123	− 1.732532E−1
40	3.568411	− 1.427551E−1
45	3.423812	− 1.161108E−1
50	3.307426	− 9.202198E−2

Table 7 illustrates the pressure drop as a function of rising Re. The largest pressure difference is observed at *Re* = *1,* roughly 0.45548, while the smallest is observed at Re = 50, around 0.074106917.

**Table 7:** Pressure gradient with $n=1$ for various $Re.$

Re	$\delta p={p}_{2}-{p}_{1}$
1	4.55481103E−1
5	1.30816555E−1
10	9.8082045E−2
15	8.7934955E−2
20	8.2965494E−2
25	7.9992039E−2
30	7.8005690E−2
35	7.6587183E−2
40	7.5531519E−2
45	7.4726817E−2
50	7.4106917E−2

**Figure 4:**
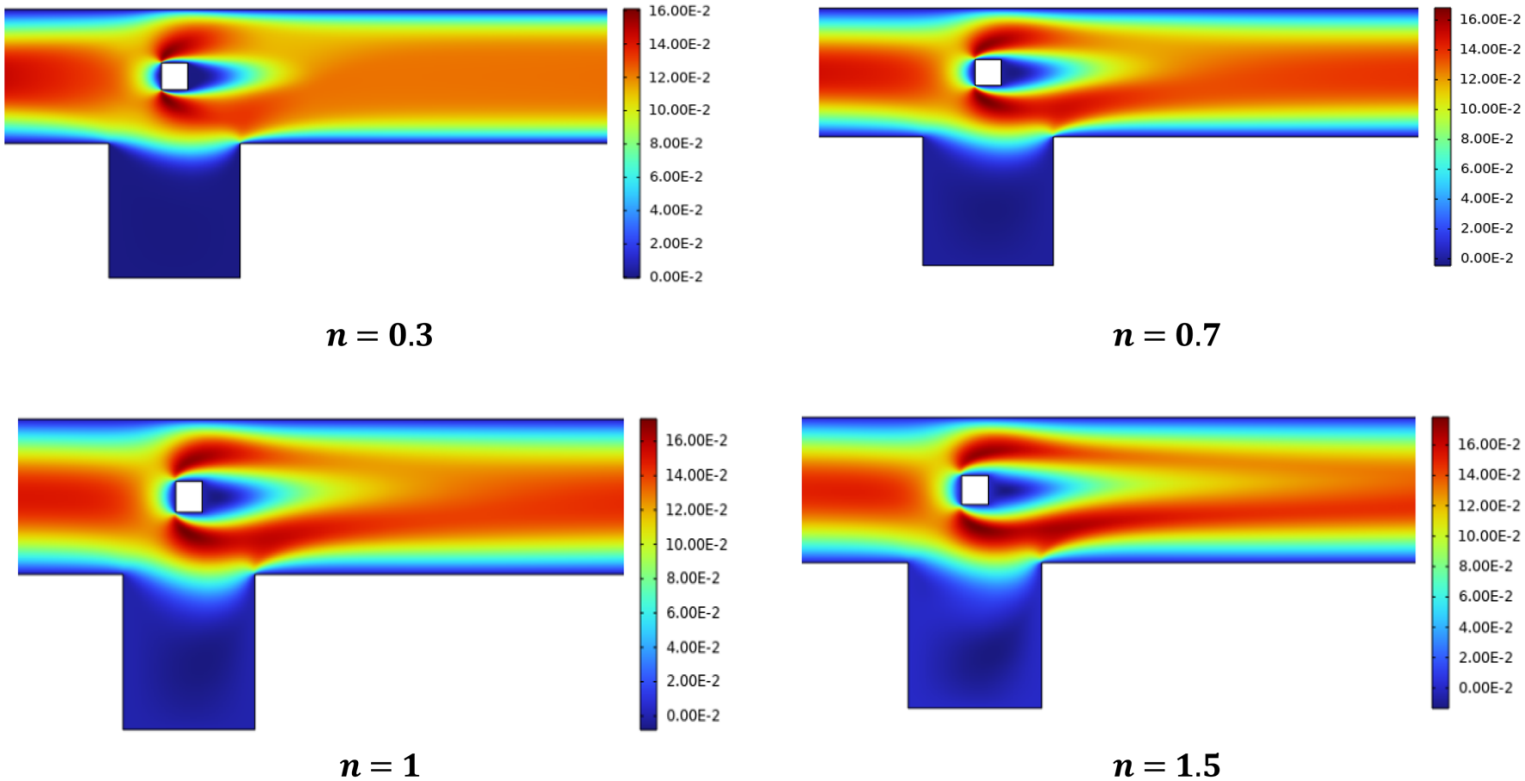
velocity profile at Re = 20 for various n.

The velocity distribution is brought into focus in Fig. 4 by adjusting the n between 0.3 and 1.5. The Power law flows with *n* = 0.5 exhibit shear thinning behaviour, while those with *n* = 1 behave like Newtonian fluids and for *n* = 1.3 and 1.5 behave like shear thickening fluids. Since the parabolic velocity is induced at the inlet, and the other boundaries are maintained at no slip conditions, the change in velocity that is only detected is near barriers and other parts of the channel driven cavity. During power law fluid flow in the cavity region, additional circulating flow is generated, and vortices occur.

**Figure 5:**
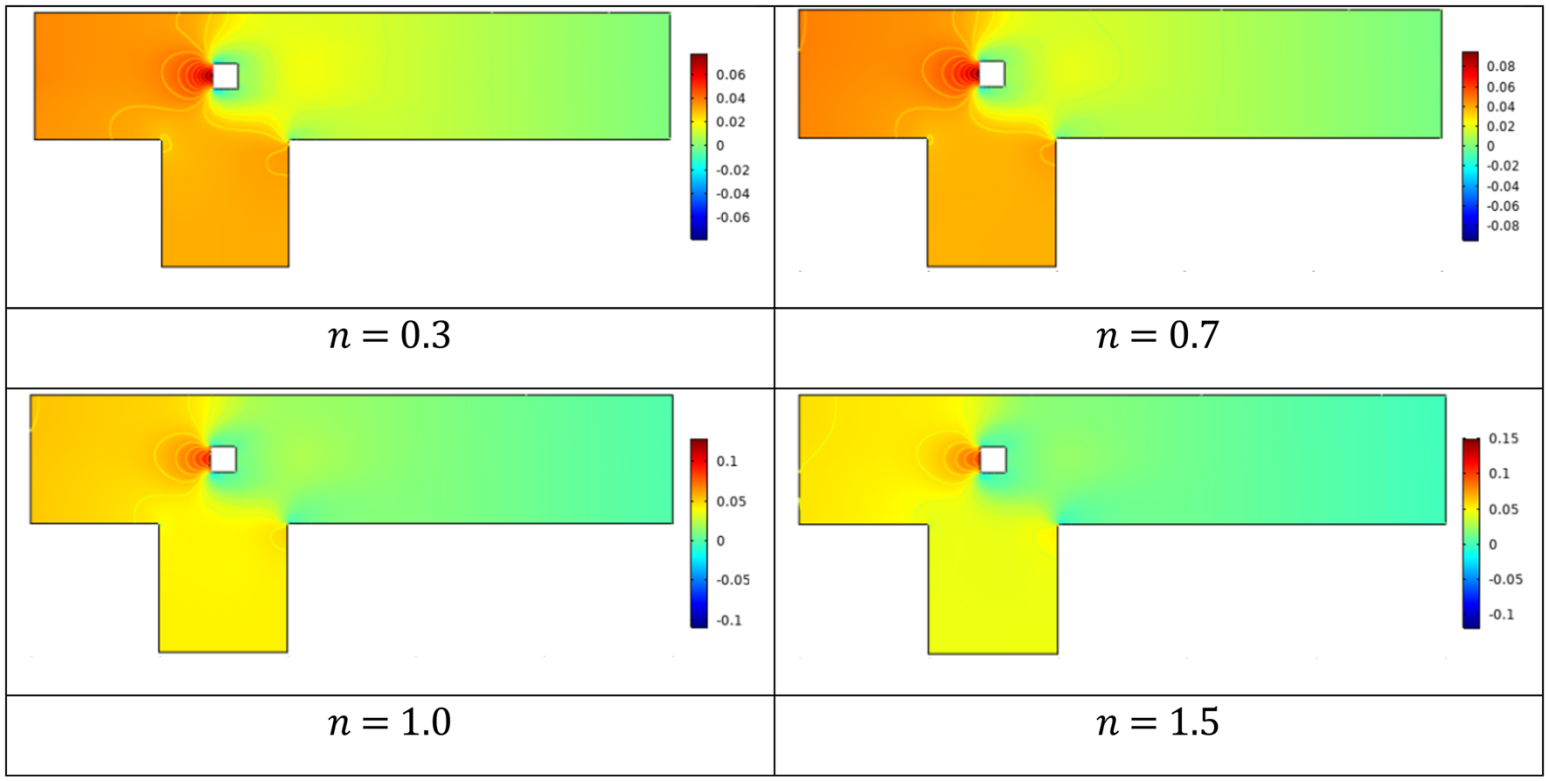
Pressure distribution for several n.

**Figure 6:**
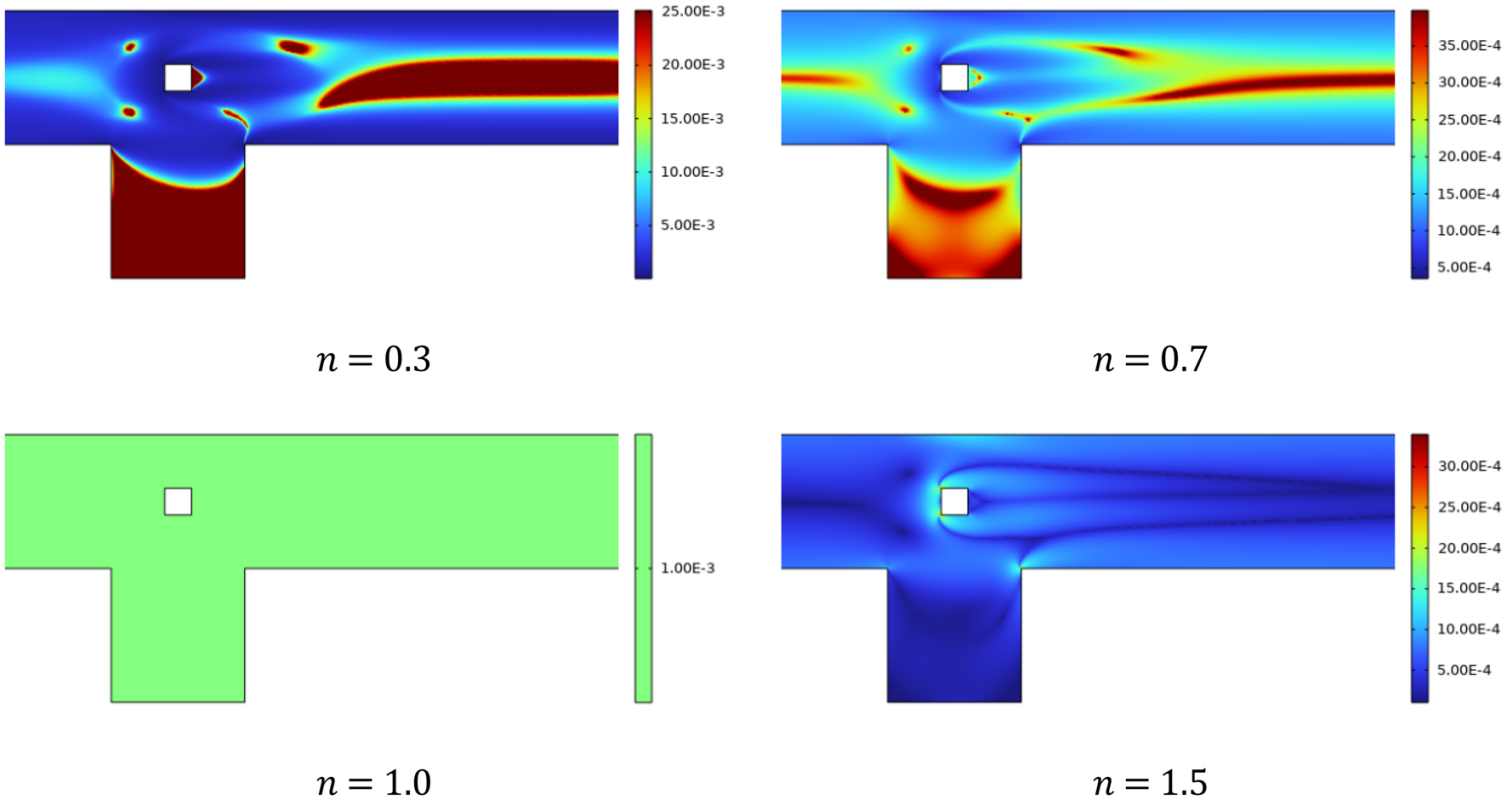
Viscosity plots at Re = 20 for various n.

Examining the effect of increasing *n* on the pressure gradient in a channel-driven cavity at *Re* = 20 is shown in Fig. 5. It is revealed that when n increases, the power law fluid transforms from a shear thinning state to a Newtonian state, and finally to a shear thickening state. Since the viscosity of the fluid increases and the velocity of the fluid decreases as *n* grows larger, less force is applied to the barrier as *n* grows larger. As a result, the injected fluid displays higher pressures for cases where n is greater than one, also known as shear thickening case. For shear-thinning cases stagnation pressure is reduced. Figure 6 depicts viscosity plots showing the yielded and unyielded zones for a variety of scenarios, which offers more comprehension of the flow behaviour activity. The shear thickening process results in the formation of small zones that are not yielding, which is an indication that the flow is weak because of the influence of fluid yield stress. Thus, liquid-like behaviour is promoted by decreasing values of n during shear thinning, whereas it is inhibited by the fluid yield stress. For the case *n* = 1, the viscosity is constant throughout the domain which confirms the Newtonian flow regime. Some islands of viscosity also revealed in the vicinity of obstacle for shear-thinning cases n < 1.

Table 8 depicts the two-performance metrics Mean Square Error (MSE) and the coefficient of determination (R) for ${C}_{D}$ and ${C}_{L}$ at various stages of the developed neural network. The MSE for all cases is approaching zero and R value is close to 1 showing higher predictivity of fluid forces via the established net**.**

**Table 8:** Statistical error analysis of ${C}_{D}$ and ${C}_{L}$ for different $n.$

${C}_{D}$	$n=0.7$		$n=1.0$		$n=1.3$	
	MSE	R	MSE	R	MSE	R
Training	2.56E−05	9.96E−01	2.65E−06	9.99E−01	3.29E−04	9.98E−01
Validation	1.99E−05	9.93E−01	3.12E−06	9.99E−01	2.89E−04	9.95E−01
Testing	2.18E−05	9.94E−01	2.56E−06	9.98E−01	3.09E−04	9.86E−01
${C}_{L}$	MSE	R	MSE	R	MSE	R
Training	6.15E−07	9.92E−01	2.76E−08	9.98E−01	8.13E−05	9.97E−01
Validation	5.59E−07	9.89E−01	2.25E−09	9.99E−01	4.68E−05	9.37E−01
Testing	5.73E−07	9.99E−01	4.35E−08	9.98E−01	6.55E−05	9.75E−01

**Figure 7:**
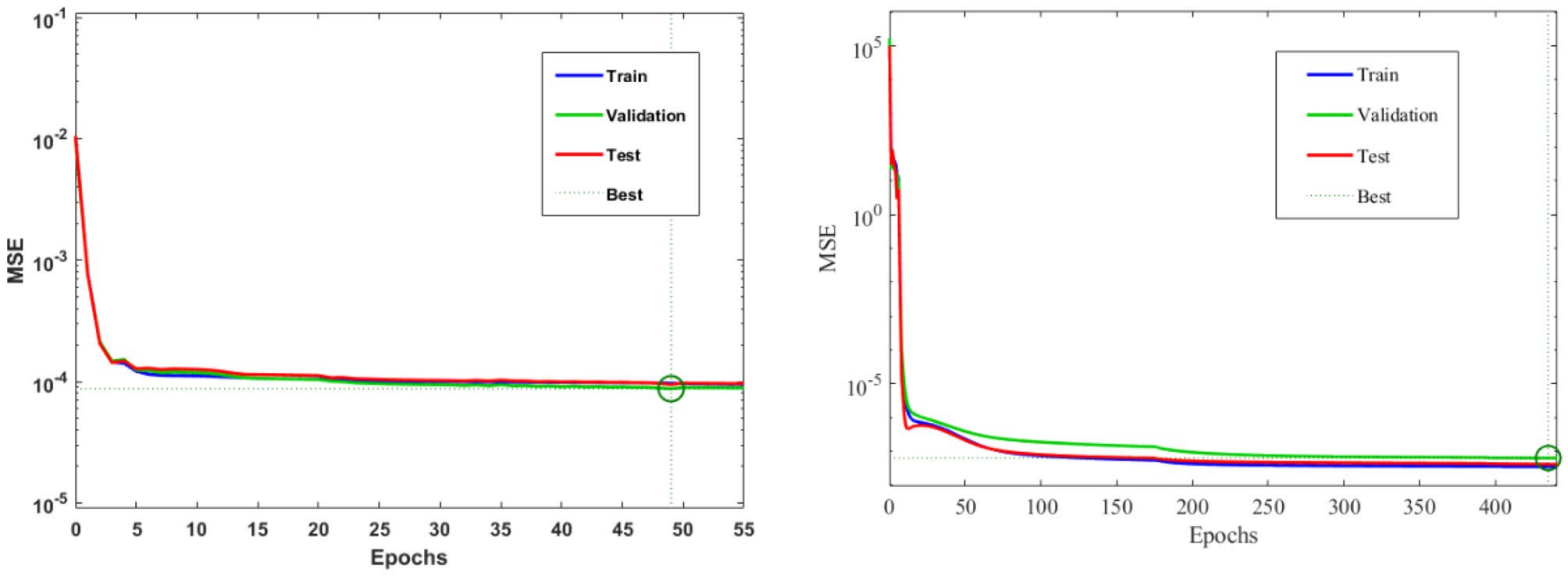
MSE for ${C}_{D}$ (left) and ${C}_{L}$ (right) for $n=0.7.$

The goodness of fit is shown in Fig. 7 for training, testing and validation phases. This fitted regression line covers most of the data points as is evident from the *R* values. For the sake of brevity only one case for $n=0.7$ has been shown here both for ${C}_{D}$ and ${C}_{L}$. The convergence of Loss function i.e., MSE versus the number of epochs is presented in Fig. 7. It is observed that a smaller number of epochs required for the convergence of loss function for ${C}_{D}$ as compared with ${C}_{L}$.

**Figure 8:**
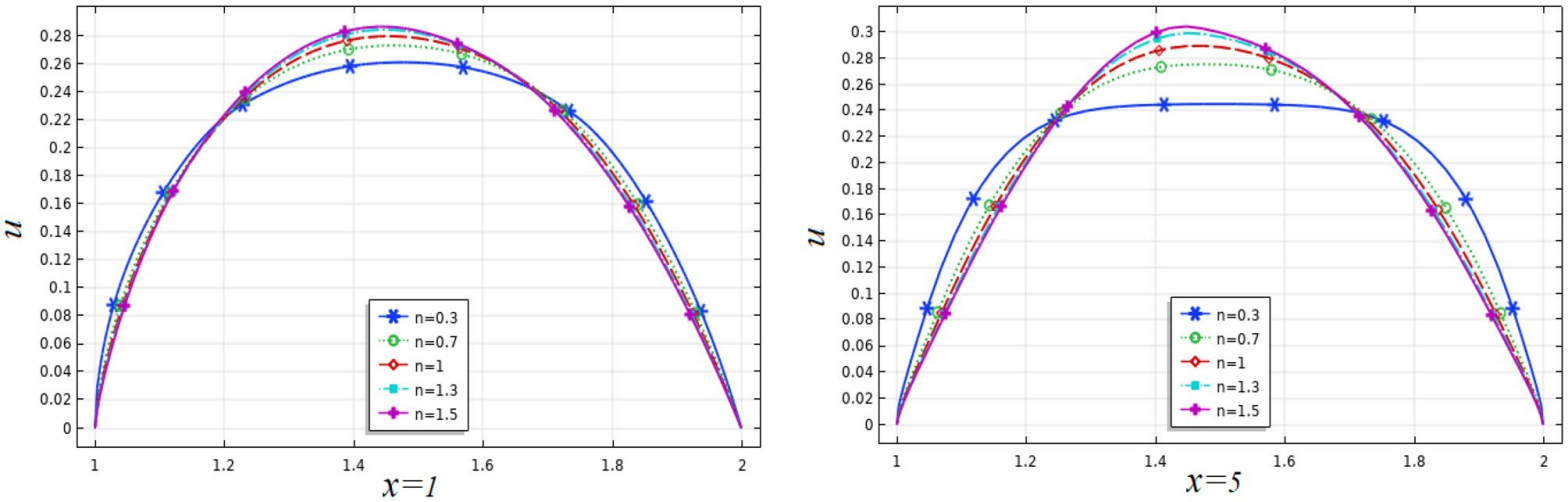
Line graphs of velocity for various $n.$

In Fig. 8, we see what happens to the flow's velocity at *x* = 1 (before obstacle) and at *x* = 5 (the outlet) by generating line graphs. Because of the generation of a completely developed flow in this stream, the perfectly parabolic behavior is observed at *n* = 1 and for other values of *n* the parabola is flattened and sharpened in the center of the channel representing the shear rate dependence of viscosity and consequently on velocity.

### Time dependent case *Re* = 100

**Figure 9:**
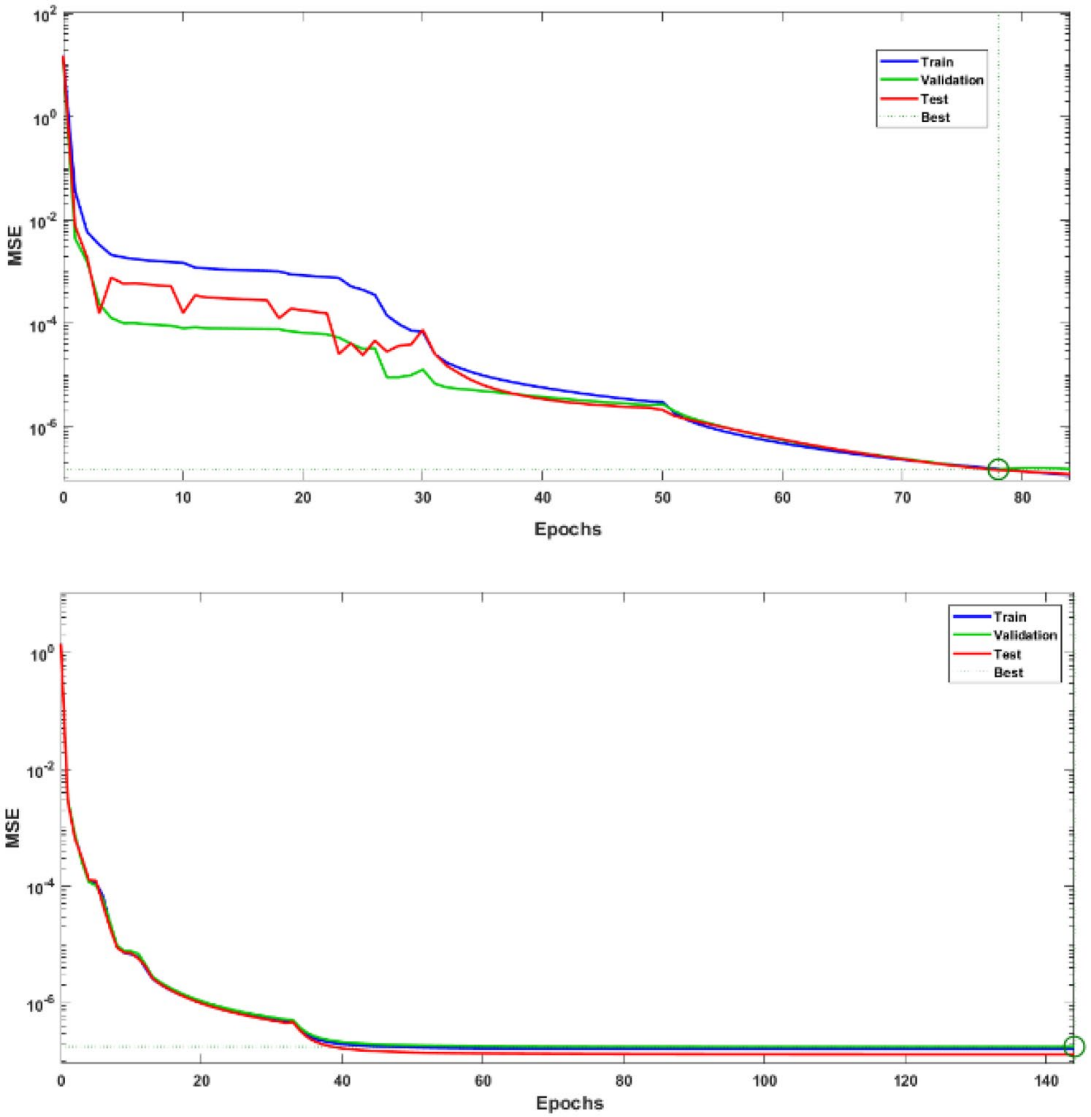
MSE analysis of ${C}_{D}$ for *n* = 0.7 and 1.3.

**Figure 10:**
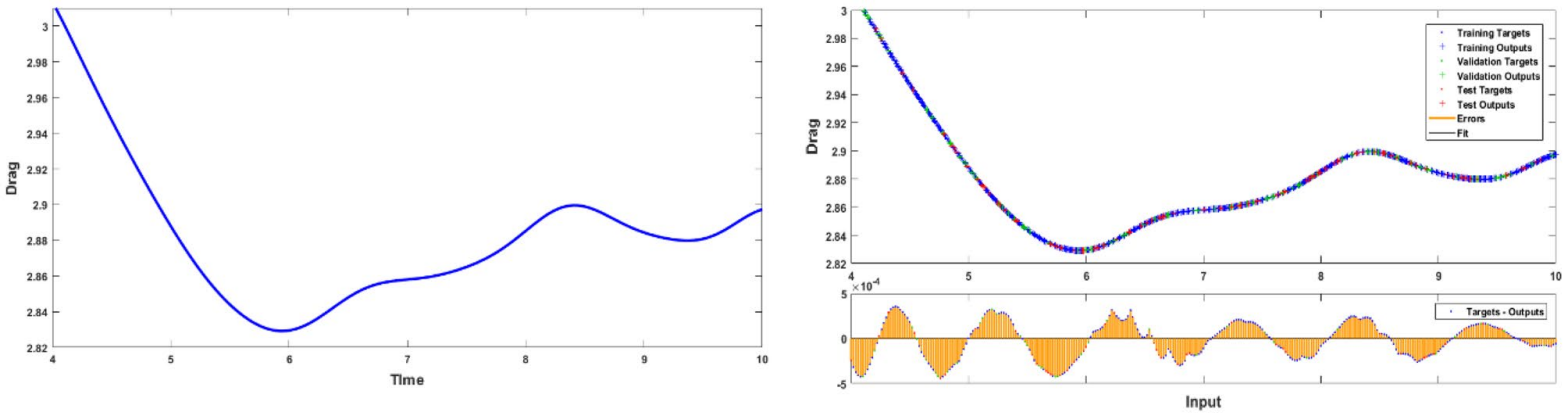
Fitness analysis of $C_{D}$ for $n = 0.5$.

**Figure 11:**
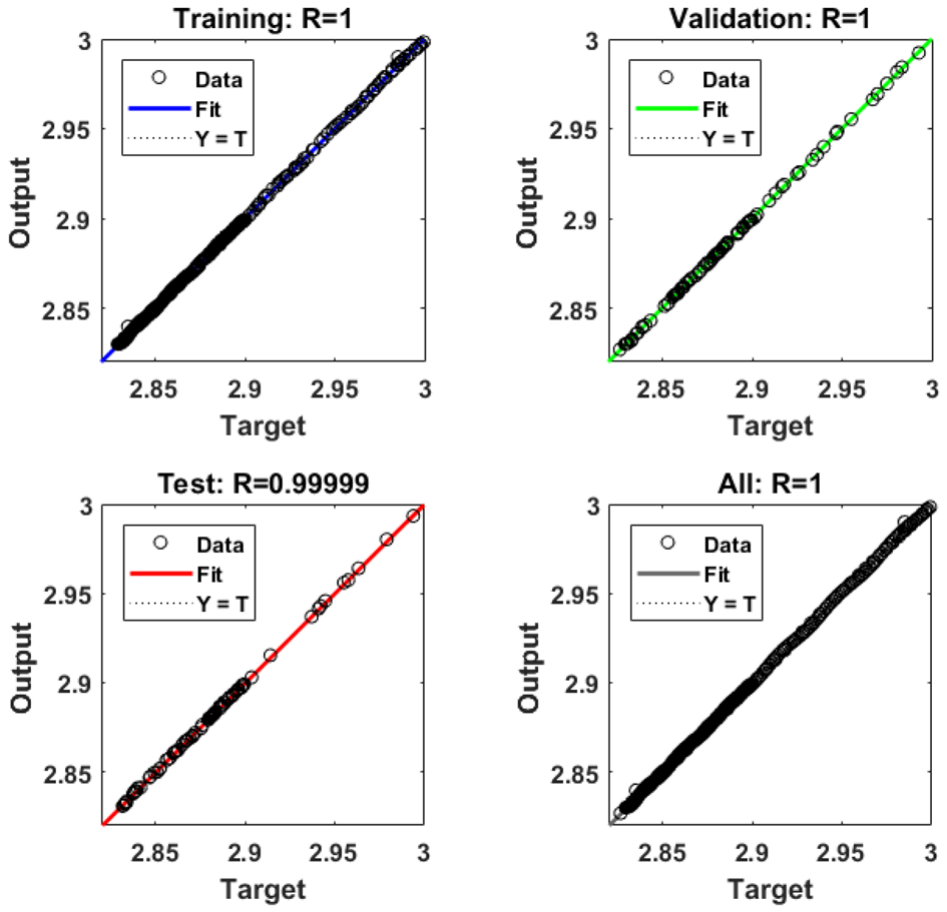
Fitness analysis of $C_{D}$ for $n = 0.5$.

Having obtained promising results for the stationary case, we extended the study to a time dependent case while increasing the Reynolds number to 100. Simulations have been run for $$t \in [0,10]{\text{ with }}\Delta t = 0.001[0,10]$$$${\text{ with }}\Delta t = 0.001$$ producing 1000 data points for each case. From the obtained values, 70% data was used for training phase, 15% for testing and 15% for validation phase. Figure 9 shows the Levenberg–Marquardt neural network training efficiency plots. Because the MSE values have decreased, the solution appears to be more reliable. From relatively high levels at the beginning of the training phase to lower values as the epochs progress, these graphs depict the progressive decline in MSE values that occurs over the course of the training term. Extensive epochs for *n* = 0.7 and *n* = 1.3 are included in the analysis, which includes validation, testing, and training stages. Appropriate models for error analysis displayed were shown in Fig. 10. The neural network is led by this graph, which is responsible for orchestrating the process of learning patterns and relationships within the data that is presented. These graphs reduce the discrepancy between the outcomes that were predicted and those that really occurred by altering the parameters of the network in an iterative manner. This helps to improve the model's ability to generalize data that has not been seen before. Regression plots measure the degree of association between outcomes and objectives. A stronger relationship is indicated by a value of R that is close to 1, while a value of R that is close to 0 indicates that the association is arbitrary. In Fig. 11, plots of FEM-Net are displayed in comparison to the reference CFD solution for a value of n equal to 0.5.

Finally, we show the efficiency of hybrid FEM-ANN approach by comparing the computational time for the calculation of drag and lift coefficients using CFD first and then predicting these force coefficients through ANN approach without CFD. Table 9 represents such a comparison. One can notice a drastic reduction in the computational time while predicting drag and lift through ANN approach. This data is collected by running CFD and ANN tool using a system with Intel^®^ Core™ i5 processors.

**Table 9:** Computational time comparison of CFD versus ANN approach for Re = 100.

$n$	CFD computation time	ANN time
$0.5$	$154 {\text{min}}$	$15 {\text{min}}$
$1.0$	$125 {\text{min}}$	$12 {\text{min}}$
$1.5$	$98 {\text{min}}$	$10 {\text{min}}$

## Conclusions

To forecast drag and lift coefficients with fully unknown data, a neural network has been trained and validated using findings from Finite Element based CFD simulations. As a first phase of this hybrid approach, the training and validation data sets for drag and lift coefficients have been generated by CFD and then are fed through ANN with optimal number of neurons and inner layers. A well-known feed-forward back-propagation LM algorithm, which offers second order training speed, was utilized to train the network.

We have shown that a coupled CFD-ANN approach can lead to a drastic reduction in computational resources in terms of memory and time considerations. This hybrid approach can accelerate the convergence of overall scheme. Numerical test cases have been performed in a channel driven cavity domain containing an obstacle that interacts with the flow of a fluid governed by a non-linear rheological relation. As a result of this interaction fluid forces are generated and a pressure drop at the front and back end of the obstacle. These quantities of interest have been computed via CFD simulations and served as the basis for training and validation data sets for artificial neural networks. The key outcomes of this study are listed below.

i. The ANN approach is much less computationally expansive than performing the CFD simulations for all values of *n* for time dependent case.
ii. More epochs noticed in the training phase for lift coefficient as compared with the training phase of drag coefficient.
iii. The agreement between the CFD results and the data predicted from ANN determined via the correlations is within less than ± 5% errors.
iv. ${C}_{L}$ with negative values are achieved when upward pressures are greater than those acting downward, while ${C}_{L}$ with positive values are achieved when downward forces are greater.
v. In the case of a Power law fluid with shear thinning, the ${C}_{D}$ and ${C}_{L}$ have a smaller magnitude in contrast to the shear thickening version of the fluid.
vi. The Re has a characteristic that decreases when applied to the pressure constraint difference in the vicinity of a square obstruction.
vii. As the *n* rises, the velocity profile becomes peaky in the center, and flattening otherwise that truly reflects the features of PL fluids.

It has been shown that data-driven techniques are appropriate for fluid dynamics problems, and it has been determined that ANN is a trustworthy instrument that efficiently lowers the cost of CFD simulations. This approach in future will be applied to 3D and turbulent flows where advantages would be more prominent.

## Data Availability

Data will be available on request by contacting the corresponding author, Dr. Ahmed Refaie Ali, via ahmed.refaie@science.menofia.edu. e.g., OR via Dr. Afraz Hussain Majeed at afraz@ujs.edu.cn.
